# Higher incidence of perineal community acquired MRSA infections among toddlers

**DOI:** 10.1186/1471-2431-11-96

**Published:** 2011-10-27

**Authors:** Alexis C McCullough, Melissa Seifried, Xiaochen Zhao, Jeffrey Haase, William J Kabat, Ram Yogev, Robert M Blumenthal, Deepa Mukundan

**Affiliations:** 1 The University of Toledo College of Medicine, 3000 Arlington Avenue, Toledo-OH, 43614, USA; 2 Department of Pediatrics, Division of Infectious Diseases, The University of Toledo College of Medicine and Mercy Children's Hospital, 2222 Cherry Street, Toledo-OH, 43608, USA; 3 Mercy Integrated Laboratories, 2222 Cherry Street, Toledo-OH, 43608, USA; 4 Department of Pediatrics, Division of Infectious Diseases, Children's Memorial Hospital, 2300 Children's Plaza, Chicago-IL 60614-3363, USA; 5 Department of Medical Microbiology and Immunology, The University of Toledo College of Medicine, 3000 Arlington Avenue, Toledo-OH, 43614, USA; 6 Program in Bioinformatics & Proteomics/Genomics, The University of Toledo College of Medicine, 3000 Arlington Avenue, Toledo-OH, 43614, USA

## Abstract

**Background:**

A six-fold increase in pediatric MRSA infections, prompted us to examine the clinical profile of children with MRSA infections seen at Mercy Children's Hospital, Toledo, Ohio and to characterize the responsible strains.

**Methods:**

Records were reviewed of pediatric patients who cultured positive for MRSA from June 1 to December 31, 2007. Strain typing by pulsed field gel electrophoresis (PFT) and DiversiLab, SCC*mec *typing, and PCR-based *lukSF-PV *gene (encodes Panton-Valentine leukocidin), arginine catabolic mobile element (ACME) and *cap*5 gene detection was performed.

**Results:**

Chart review of 63 patients with MRSA infections revealed that 58(92%) were community acquired MRSA (CAMRSA). All CAMRSA were skin and soft tissue infections (SSTI). Twenty five (43%) patients were aged < 3 yrs, 19(33%) aged 4-12 and 14(24%) aged 13-18. Nineteen (76%) of those aged < 3 yrs had higher incidence of perineal infections compared to only 2(11%) of the 4-12 yrs and none of the 13-18 yrs of age. Infections in the extremities were more common in the older youth compared to the youngest children. Overall, there was a significant association between site of the infection and age group (Fisher's Exact p-value < 0.001). All CAMRSA were USA300 PFT, clindamycin susceptible, SCC*mec *type IVa and *lukSF-PV gene *positive. Nearly all contained ACME and about 80% were *cap*5 positive. Of the 58 USA300 strains by PFT, 55(95%) were also identified as USA300 via the automated repetitive sequence-based PCR method from DiversiLab.

**Conclusions:**

CAMRSA SSTI of the perineum was significantly more common among toddlers and that of the extremities in older children. The infecting strains were all USA300 PFT. Further studies are needed to identify the unique virulence and colonization characteristics of USA300 strains in these infections.

## Background

*Staphylococcus aureus *(*S. aureus*) is a common human commensal organism and a clinically important invasive pathogen. Methicillin resistant *S. aureus *(MRSA) remains one of the most prevalent pathogens isolated from hospital patients. However, MRSA infections are increasingly arising outside of healthcare settings among individuals in the community with no established risk factors. Furthermore, the incidence of invasive community acquired MRSA (CAMRSA) disease in previously-healthy children has been increasing [1-3].

**Figure 1:**
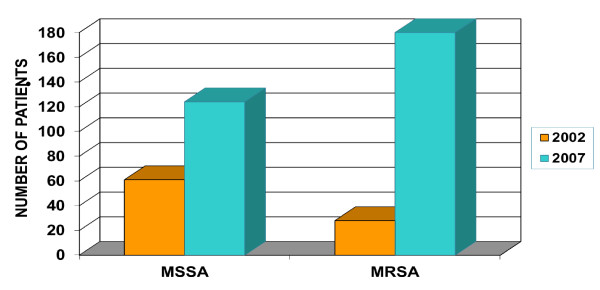
**Comparison of Pediatric MRSA Incidence in two time periods five years apart: June 1st to Dec 31st 2002 and 2007 (p < 0.0001)**.

CAMRSA strains as defined by the Centers for Disease Control and Prevention (CDC) clinical criteria [4] have some general characteristics that differentiate them from healthcare associated MRSA (HAMRSA) strains, including the presence of the Staphylococcal chromosomal cassette - SCC*mec *type IVa that confers methicillin resistance, *lukSF-PV *genes that codes for Panton-Valentine leukocidin (PVL), arginine catabolic mobile element (ACME) element that contributes to skin colonization [5], antibiotic resistance patterns [3], and pulsed field types (PFT) [6]. Several reports now show the migration of these CAMRSA strains into the hospital setting [7-9]. A six-fold increase in the number of MRSA infections among children between 2002 and 2007 (p < 0.0001) (Figure 1) prompted us to examine both the clinical profile of the patients, and the molecular profile of the infecting strains at Mercy Children's Hospital Toledo, Ohio. We also compared two strain typing methods - pulsed field typing (PFT) and the automated rapid typing method using repetitive sequence-based PCR, the DiversiLab system [10].

## Methods

### MRSA isolate collection and patient medical record review

This is a retrospective, descriptive, single-cohort study conducted at Mercy Children's Hospital, Toledo, Ohio and was approved by the institutional review board. Pediatric patients (< 18 yrs of age), who were culture positive for MRSA between June 1 to December 31, 2007, were identified by the clinical microbiology laboratory (Mercy Integrated Laboratories, Toledo, Ohio). This facility also provided us with a sample of the clinical isolate that had been frozen at -80°C in a solution of 50% brain-heart infusion and 50% glycerol for later analysis.

Medical charts of these patients were reviewed for social and demographic information including age, gender, race, socio-economic status based on insurance, pre-existing history of skin and soft tissue infection (SSTI); respiratory, cardiovascular, gastrointestinal, and nervous system diseases, type of care (outpatient *vs*. emergency room *vs*. inpatient care) and site of infection. Antimicrobial susceptibilities were determined based on Clinical and laboratory Standard Institute (CLSI) guidelines at the clinical laboratory using the Vitek system for oxacillin, erythromycin, clindamycin, vancomycin, ciprofloxacin, tetracycline, trimethoprim/sulfamethoxasole, rifampin and linezolid. All erythromycin-resistant strains were tested for inducible clindamycin resistance using the D-test [11] before final susceptibilities were reported.

### Strain Typing

*Pulsed Field typing (PFT)*: Strain typing was done by PFT as described by Chang *et al *[12], using *Sma*I as the restriction enzyme, at the Children's Memorial Hospital, Chicago, Illinois. The relatedness of isolates was based on visual comparison of band patterns by the use of criteria described by Tenover *et al *[13].

*DiversiLab *typing: Strain typing was also performed using the DiversiLab System at Mercy Integrated Laboratories. This is an automated method using repetitive sequence-based PCR that targets multiple noncoding repetitive sequences in the genomic DNA. Band patterns were subsequently analyzed using the web-based DiversiLab software [10].

### DNA Extraction and PCR

Clinical isolates were grown on 5% blood agar plates overnight at 37°C. DNA was extracted from the isolates using the Wizard^® ^Genomic DNA Purification Kit. PCR amplification was then performed on all patient isolates for the presence of *mecA*, SCC*mec *typing, *arcA*, ACME, *lukSF-PV *and *cap5 *genes (Additional file 1) [14-17].

### Statistical Analysis

Fisher's Exact test was used to explore for associations of site of infection, pre-existing SSTI and respiratory disease with age group and PFGE type. A Fisher's Exact p-value of < 0.05 was considered statistically significant. Data were analyzed using SAS (SAS Cary, NC, version 9).

## Results

From June 1, 2007 to December 31, 2007, 63 pediatric patients with MRSA infections were seen at the emergency room, outpatient clinics and the inpatient ward at Mercy Children's Hospital. Of these, only 5 patients did not meet the CDC clinical criteria for CAMRSA [4]. Thus, 58 (92%) of all pediatric MRSA infections were community-acquired. All of these CAMRSA infections were SSTIs. During this time period there were no other culture positive invasive pediatric MRSA infections like bacteremia, sepsis, endocarditis, pneumonia, or osteomyelitis.

The patient characteristics for CAMRSA infections are shown in Table 1. The turnover of patients at Mercy Children's Hospital in 2007 was about 22,000 (inpatients 2400) pediatric patients. In 2007, 26% of all children seen at Mercy Children's hospital were African American compared to 62% of our CAMRSA patients and; 51% were Medicaid insurance patients and therefore belonged to the lower socio economic group compared to 74% of our CAMRSA patients. All of these CAMRSA isolates were susceptible to trimethoprim/sulfamethoxasole, clindamycin, rifampin, linezolid and vancomycin.

**Table 1:** Patient Characteristics

	n (%)
Age group (years)	
0-3	25 (43%)
4-12	19 (33%)
13-18	14 (24%)

Gender	
Female	36 (62%)
Male	22 (38%)

Race	
Caucasian	16 (29%)
African American	34 (62%)
Other	5 (9%)

Type of Visit	
ER	27 (47%)
Inpatient	11 (19%)
Outpatient	20 (34%)

Insurance	
Private	6 (11%)
Medicaid	43 (74%)
Uninsured	9 (16%)

Preexisting Conditions *(patients may have more than 1)*	
SSTI	24 (41%)
Respiratory Disease	22 (38%)
Gastrointestinal	9 (16%)
Cardiovascular	3 (5%)
Nervous system	1 (2%)
None	19 (33%)

Site of Infection	
Face	4 (7%)
Lower extremity	18 (31%)
Perineal	21 (36%)
Trunk	4 (7%)
Upper extremity	11 (19%)

ER: Emergency room, SSTI: Skin and soft tissue infection

The distribution of SSTI sites is shown in Table 2 and reflects an age associated shift. In children ages 0-3 years, 19 (76%) had perineal SSTI which was more common. In contrast, only 2 (11%) of the children ages 4-12 had perineal SSTI and none of the 13-18 year olds. Infections in the extremities were more common in the older youth compared to the youngest children: 3 (12%) in ages 0-3, 13 (68%) in ages 4-12, and 13 (93%) in ages 13-18. Overall, there was a significant association between site of the infection and age group (Fisher's Exact p-value < 0.001) (Table 2).

**Table 2:** Associations with Age Group

	Age 0-3 N = 25	Age 4-12 N = 19	Age 13-18 N = 14	Fisher's Exact Two-tailed P-value
**Site of Infection**				< 0.001
Face	2 (8%)	1 (5%)	1 (7%)	
Lower extremity	2 (8%)	8 (42%)	8 (57%)	
Perineal	19 (76%)	2 (11%)	0 (0%)	
Trunk	1 (4%)	3 (16%)	0 (0%)	
Upper extremity	1 (4%)	5 (26%)	5 (36%)	

**Respiratory disease pre-existing condition**	7 (28%)	9 (47%)	6 (43%)	0.42

**SSTI pre-existing condition**	12 (48%)	9 (47%)	3 (21%)	0.25

**PFT Sub-type 1 (compared to types 2-7 combined)**	19 (79%)	14 (74%)	9 (64%)	0.59

PFT: Pulse field type, SSTI: Skin and soft tissue infection

**Figure 2:**
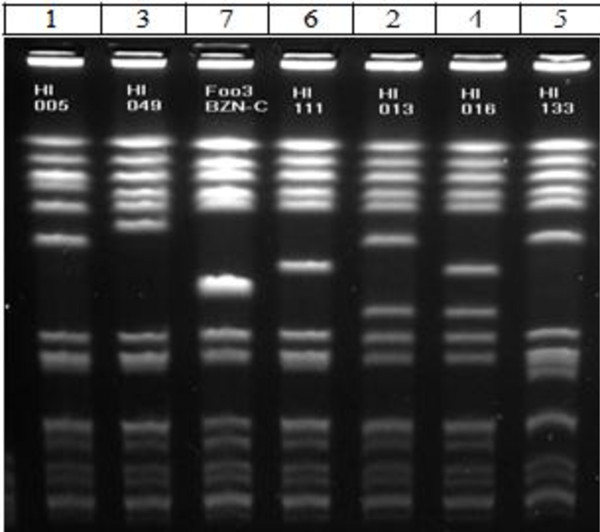
**USA300 PFT subtypes**. This is a picture of the USA300 strains all run on a single gel and are labeled according to the PFT subtypes in Table 2, 3 & 4.

Nineteen (33%) children had no pre-existing condition. Age group was not associated with having either respiratory disease or SSTI as a pre-existing condition (Fisher's Exact p-values > 0.05 - Table 2). Pulsed field typing (*Sma*l PFT) of the 58 CAMRSA isolates revealed that they were all USA300 PFT with seven different subtypes (Figure 2), and one sub-type dominated in 72% (n = 42) (Table 3). PFGE sub-type I (compared to sub-types 2-7) was not associated with site of infection or with having respiratory disease or SSTI as a pre-existing condition, all with Fisher's Exact p-values > 0.05 (Table 4). The DiversiLab typing of the 58 CAMRSA strains revealed that 55 (95%) were indeed USA300 PFT while 3 strains were identified as USA500.

**Table 3:** Molecular epidemiology of USA300 PFT strains (n = 58)

USA300 PFT subtypes n(%)	Diversilab type* n(%)	*mec*A	SCC*mec *type	ACME	*lukSF-PV *	*cap*5
**1 - **42(72%)	USA300-41(98%) USA500-1(24%)	42	IVa-42	42	42	30(71%)

**2 **- 6(10%)	USA300-4(67%) USA500-2(33%)	6	IVa-6	6	6	6

**3 **- 4(7%)	USA300-4	4	IVa-4	4	4	4

**4 **- 2(3%)	USA300-2	2	IVa-2	1	2	2

**5 **- 2(3%)	USA300-2	2	IVa-2	2	2	2

**6 **- 1(2%)	USA300-1	1	IVa-1	0	1	1

**7 **- 1(2%)	USA300-1	1	IVa-1	1	1	1

**Total(n)**	**58**(100%)	**58**(100%)	**58**(100%)	**56**(97%)	**58**(100%)	**46**(79%)

PFT: Pulsed field type; SCC: Staphylococcal Cassette Chromosome, *lukSF-PV *genes that encode Panton-Valentine leukocidin (PVL), ACME: Arginine catabolic mobile element.

*The DiversiLab strain types are named as corresponding PFT's based on a standardized comparison.

For USA300 PFT subtypes please see figure 2.

**Table 4:** Associations with PFT Sub-type

	PFT Sub-type 1 N = 42	PFT Sub-types 2-7 N = 16	Fisher's Exact Two-tailed P-value
**Site of Infection**			
Face	2 (5%)	2 (13%)	0.35
Lower extremity	11 (26%)	7 (43%)	
Perineal	16 (38%)	5 (31%)	
Trunk	4 (10%)	0 (0%)	
Upper extremity	9 (21%)	2 (13%)	

**Respiratory disease pre-existing condition**	15 (36%)	7 (47%)	0.54

**SSTI pre-existing condition**	18 (43%)	6 (40%)	1.0

PFT: Pulse field type, SSTI: Skin and soft tissue infection

All 58 CAMRSA strains were *mecA and lukSF-PV *positive, with SCC*mec *type IVa; 56 (97%) contained ACME and the *cap5 *gene was present in 46 (79%) (Table 3). We observed that all the *cap5 *negative strains belonged to a single USA300 PFT pattern (Table 3 and Figure 2).

## Discussion

Figure 1 clearly demonstrates a six fold increase in the incidence of pediatric MRSA infections from 2002 to 2007 (p < 0.0001). A clear majority of our pediatric MRSA infections were CAMRSA, which is consistent with the trend reported for the United States [18] and Europe [19,20].

A significant association of age with site of infection has for the first time been demonstrated in our study. Perineal MRSA colonization in children has been recorded in the daycare setting [21]. Koski *et al *indicate that pediatric perineal infections with MRSA are increasing [22]. We found that such infections were more common to the 0-3 y-old cohort. This age preference could be due to increased rates of perineal CAMRSA colonization in this age group, but may also reflect use of diapers and possible dermabrasion caused by vigorous wiping of the area during diaper changes or both. Transfer of vancomycin resistance gene *van*A from vancomycin resistant *Enterococcus *to MRSA has been shown to occur in vitro and in vivo [23]. Perineal CAMRSA infection in children could contribute to the emergence of vancomycin resistant *S.aureus *strains when co-colonized with vancomycin resistant *Enterococcus *[24].

Of interest, all of the CAMRSA strains in our study were susceptible to clindamycin, contrary to higher rates of resistance reported for Alaska, Houston and San Francisco [25-27].

Strain typing of our patients' MRSA isolates supports the observation that USA300 PFT is the most common causative strain and is clonal [27]. The molecular characteristics of our isolates were similar to the USA300 strains from other published reports in that they were SCC*mec *type IVa and *lukSF-PV *positive [27,28]. 56/58 of the strains were also positive for ACME, a novel mobile genetic region predominantly reported in USA300 MRSA strains that potentially enhance colonization and virulence [29]. We observed that all the 12 strains that were *cap5 *gene negative belonged to the predominant USA300 PFT subtype (Table 3 and Figure 2). *cap5 *gene encodes for a capsule that enhances the virulence of *Staphylococcus aureus*. It has been used as vaccine target. The clinical significance of *cap5 *negative strains in our study is unclear at this time.

Typing using the DiversiLab system is rapid and user friendly compared to PFT. However, differentiating PFT USA300 from USA500 is tricky using DiversiLab [30]. Of interest, 3/3 isolates identified by PFT as USA300 were mis-identified by DiversiLab as USA500. These isolates were also positive for ACME which is a mobile genetic element that is thought to be acquired by USA300 as it evolved from its progenitor USA500 [31].

## Conclusion

The incidence of CAMRSA infection is increasing in the pediatric population of Northwest Ohio. SSTIs are the most common type of infection and among children < 3 years of age with perineal SSTIs being the dominant form caused by strain USA300 PFT that carries the SCC*mec *type IVa, *lukSF-PV *gene and ACME. The automated rapid strain typing method, the DiversiLab system, is not as discriminative as PFT.

Further investigations are needed to assess the extent of USA300 perineal colonization in toddlers and to identify unique virulence characteristics to develop strategies for prevention and treatment of these infections.

## Abbreviations

ACME: Arginine catabolic mobile element; CAMRSA: Community-acquired methicillin resistant *Staphylococcus aureus*; MRSA: Methicillin resistant *Staphylococcus aureus*; PCR: Polymerase chain reaction; PFT: Pulsed field type (or) pulsed field gel electrophoresis; *S.aureus*: *Staphylococcus aureus*; SSTI: Skin and soft tissue infection.

## Competing interests

The authors declare that they have no competing interests.

## Authors' contributions

AM conducted patient chart review, ran PCR tests and wrote the first draft of the manuscript. MS did the collection and freezing of MRSA samples and ran PCR tests. XZ ran PCR tests and managed the MRSA database. JH did the DiversiLab typing. WK conducted the pulsed field gel typing experiments. RY was involved in the concept of the study and reviewed the manuscript. RB was involved in the concept of the study; contributed his expertise in conducting the PCR experiments; edited and reviewed the manuscript; and mentored medical students AM and MS in the laboratory. DM was the principal investigator and was involved in the concept, planning and coordination of the study. She also mentored medical students AM and MS. In addition, she was responsible for writing, editing and submission of the manuscript. All authors read and approved the final manuscript.

## Pre-publication history

The pre-publication history for this paper can be accessed here:

http://www.biomedcentral.com/1471-2431/11/96/prepub

## Supplementary Material

Additional file 1 **Primers used for the PCR reactions for *mecA*, SCC*mec *typing, *arcA*, ACME, *lukSF-PV *and *cap5 *genes**. Details of the sequences of all the primers used in this study along with the references are in the file. Click here for file

## References

[B1] Chambers HF The changing epidemiology of *S. aureus*? Emerg Infect Dis 2001 7 178 82 10.3201/eid0702.010204 11294701 PMC2631711

[B2] Zetola N Francis JS Nuermberger EL Bishai WR CAMRSA: An emerging threat Lancet Infect Dis 2005 5 275 86 10.1016/S1473-3099(05)70112-2 15854883

[B3] Shapiro A Raman S Johnson M Piehl M CAMRSA infections in North Carolina children: prevalence, antibiotic sensitivities, and risk factors N C Med J 2009 70 102 7 19489364

[B4] Naimi TS LeDell KH Como-Sabetti K Borchardt SM Boxrud DJ Etienne J Comparison of community- and health care-associated MRSA infection JAMA 2003 290 2976 84 10.1001/jama.290.22.2976 14665659

[B5] Goering RV McDougal LK Fosheim GE Bonnstetter KK Wolter DJ Tenover FC Epidemiologic distribution of the ACME among selected methicillin-resistant and methicillin-susceptible *S.aureus isolates* J Clin Microbiol 2007 45 1981 4 10.1128/JCM.00273-07 17409207 PMC1933090

[B6] King MD Humphrey BJ Wang YF Kourbatova EV Ray SM Blumberg HM Emergence of CAMRSA USA 300 clone as the predominant cause of SSTI Ann Intern Med 2006 144 309 17 16520471 10.7326/0003-4819-144-5-200603070-00005

[B7] Chua K Laurent F Coombs G Grayson ML Howden BP Antimicrobial resistance: Not community-associated methicillin-resistant Staphylococcus aureus (CA-MRSA)! A clinician's guide to community MRSA - its evolving antimicrobial resistance and implications for therapy Clin Infect Dis [Research Support, Non-U.S. Gov't Review] 2011 52 1 99 114 21148528 10.1093/cid/ciq067

[B8] Reygaert W Methicillin-resistant Staphylococcus aureus (MRSA): prevalence and epidemiology issues Clin Lab Sci 2009 22 2 111 4 19534445

[B9] Moore CL Hingwe A Donabedian SM Perri MB Davis SL Haque NZ Comparative evaluation of epidemiology and outcomes of methicillin-resistant Staphylococcus aureus (MRSA) USA300 infections causing community- and healthcare-associated infections Int J Antimicrob Agents. [Comparative Study] 2009 34 2 148 55 10.1016/j.ijantimicag.2009.03.004 19394801

[B10] Shutt CK Pounder JI Page SR Schaecher BJ Woods GL Clinical evaluation of the DiversiLab microbial typing system using repetitive-sequence-based PCR for characterization of *S.aureus strains* J Clin Microbiol 2005 43 1187 92 10.1128/JCM.43.3.1187-1192.2005 15750081 PMC1081226

[B11] Lewis JS Jorgensen JH Inducible clindamycin resistance in Staphylococci: should clinicians and microbiologists be concerned? Clin Infect Dis 200 40 280 5 10.1086/426894 15655748

[B12] Chang N Chui L A standardized protocol for the rapid preparation of bacterial DNA for pulsed field electrophoresis Diagn Microbiol Infect Dis 1998 31 275 9 10.1016/S0732-8893(98)00007-8 9597387

[B13] Tenover FC Arbeit RD Goering RV Mickelsen PA Murray BE Persing DH Interpreting chromosomal DNA restriction patterns produced by pulsed-field gel electrophoresis: criteria for bacterial strain typing J Clin Microbiol 1995 33 2233 9 7494007 10.1128/jcm.33.9.2233-2239.1995 PMC228385

[B14] Oliveira DC de Lencastre H Multiplex PCR strategy for rapid identification of structural types and variants of the mec element in MRSA Antimicrob Agents Chemother 2002 46 2155 61 10.1128/AAC.46.7.2155-2161.2002 12069968 PMC127318

[B15] Zhang K McClure JA Elsayed S Louie T Conly JM Novel multiplex PCR assay for characterization and concomitant subtyping of SCC mec types I to V in MRSA J Clin Microbiol 2005 43 5026 33 10.1128/JCM.43.10.5026-5033.2005 16207957 PMC1248471

[B16] Lina G Piemont Y Godail-Gamot F Bes M Peter MO Gauduchon V Involvement of PVL-producing *S.aureus *in primary skin infections and pneumonia Clin Infect Dis 1999 29 1128 32 10.1086/313461 10524952

[B17] Moore PC Lindsay JA Genetic variation among hospital isolates of methicillin-sensitive *S.aureus*: evidence for horizontal transfer of virulence genes J Clin Microbiol 2001 39 2760 7 10.1128/JCM.39.8.2760-2767.2001 11473989 PMC88236

[B18] Como-Sabetti K Harriman KH Buck JM Glennen A Boxrud DJ Lynfield R CAMRSA: trends in case and isolate characteristics from six years of prospective surveillance Public Health Rep 2009 124 427 35 19445419 10.1177/003335490912400312 PMC2663879

[B19] Faria NA Oliveira DC Westh H Monnet DL Larsen AR Skov R Epidemiology of emerging MRSA in Denmark: A nationwide study in a country with low prevalence of MRSA infection J Clin Microbiol 2005 43 1836 42 10.1128/JCM.43.4.1836-1842.2005 15815005 PMC1081382

[B20] Vourli S Perimeni D Makri A Polemis M Voyiatzi A Vatopoulos A CAMRSA infections in a paediatric population in Greece Euro Surveill 2005 10 78 9 16077207

[B21] Shahin R Johnson IL Jamieson F McGeer A Tolkin J Ford-Jones EL MRSA carriage in a child care center following a case of disease. Toronto Child Care Center Study Group Arch Pediatr Adolesc Med 1999 153 864 8 10437762 10.1001/archpedi.153.8.864

[B22] Koski ME DeMarco RT Brock JW Pope JC Adams MC Thomas JC Community associated methicillin resistant staphylococcal infections in a pediatric urology practice J Urol 2008 179 1098 101 10.1016/j.juro.2007.10.086 18206937

[B23] Perichon B Courvalin P VanA-type vancomycin-resistant *S.aureus* Antimicrob Agents Chemother 2009 53 4580 7 10.1128/AAC.00346-09 19506057 PMC2772335

[B24] Warren DK Nitin A Hill C Fraser VJ Kollef MH Occurrence of co-colonization or co-infection with vancomycin-resistant enterococci and MRSA in a medical intensive care unit Infect Control Hosp Epidemiol 2004 25 99 104 10.1086/502357 14994932

[B25] Diep BA Chambers HF Graber CJ Szumowski JD Miller LG Han LL Emergence of multidrug-resistant, CAMRSA clone USA300 in men who have sex with men Ann Intern Med 2008 148 249 57 18283202 10.7326/0003-4819-148-4-200802190-00204

[B26] Kaplan SL Hulten KG Gonzalez BE Hammerman WA Lamberth L Versalovic J Three-year surveillance of CAMRSA infections in children Clin Infect Dis 2005 40 1785 91 10.1086/430312 15909267

[B27] David MZ Rudolph KM Hennessy TW Boyle-Vavra S Daum RS Molecular epidemiology of MRSA, rural southwestern Alaska Emerg Infect Dis 2008 14 1693 9 10.3201/eid1411.080381 18976551 PMC2630737

[B28] Jones RN Nilius AM Akinlade BK Deshpande LM Notario GF Molecular characterization of *S.aureus *isolates from a 2005 clinical trial of uncomplicated skin and skin structure infections Antimicrob Agents Chemother 2007 51 3381 4 10.1128/AAC.01588-06 17576829 PMC2043172

[B29] Diep BA Stone GG Basuino L Graber CJ Miller A des Etages SA The ACME and SCCmec linkage: convergence of virulence and resistance in the USA300 clone of MRSA J Infect Dis 2008 197 1523 30 10.1086/587907 18700257

[B30] Library Stats Sheet: MRSA. 2008 BioMerieux Inc. BBI-019-08 http://www.biomerieux-usa.com/upload/BBI-019-08%20LSS%20MRSA%20v3-1.pdf Accessed April 6, 2010

[B31] Li M Diep BA Villaruz AE Braughton KR Jiang X DeLeo FR Evolution of virulence in epidemic community-associated methicillin-resistant Staphylococcus aureus Proc Natl Acad Sci USA 2009 106 14 5883 8 10.1073/pnas.0900743106 19293374 PMC2667066

