# Supplementary material for: Higher incidence of perineal community acquired MRSA infections among toddlers
Source: BMC Pediatr. 2011 Oct 27;11:96. doi: 10.1186/1471-2431-11-96 (PMC3216857; doi:10.1186/1471-2431-11-96)
Supplement: Additional file 1 — Primers used for the PCR reactions for mecA, SCCmec typing, arcA, ACME, lukSF-PV and cap5 genes. Details of the sequences of all the primers used in this study along with the references are in the file. [file 1471-2431-11-96-S1.DOC]

**Additional file 1**: Primers used for the PCR reactions

| **Primer Name** | **Primer Sequence (5’-3’)** | **Conc.(µM)** | **Size(bp)** | **Specificity** | **Reference** |
| --- | --- | --- | --- | --- | --- |
| *mec*A-F  *mec*A-R | TCC AGA TTA CAA CTT CAC CAG G  CCA CTT CAT ATC TTG TAA CG | 0.2 | 162 | Internal control | [14] |
| *mec* Type I-F  *mec* Type I-R | GCTTTAAAGAGTGTCGTTACAGG  GTTCTCTCATAGTATGACGTCC | 0.048 | 613 | SCC*mec* I | [15] |
| *mec* Type II-F  *mec* Type II-R | CGTTGAAGATGATGAAGCG  CGAAATCAATGGTTAATGGACC | 0.032 | 398 | SCC*mec* II | [15] |
| *mec* Type III-F  *mec* Type III-R | CCATATTGTGTACGATGCG  CCTTAGTTGTCGTAACAGATCG | 0.04 | 280 | SCC*mec* III | [15] |
| *mec* Type IVa-F  *mec* Type IVa-R | GCCTTATTCGAAGAAACCG  CTACTCTTCTGAAAAGCGTCG | 0.104 | 776 | SCC*mec* IVa | [15] |
| *mec* Type IVb-F  *mec* Type IVb-R | TCTGGAATTACTTCAGCTGC  AAACAATATTGCTCTCCCTC | 0.092 | 493 | SCC*mec* IVb | [15] |
| *mec* Type IVc-F  *mec* Type IVc-R | ACAATATTTGTATTATCGGAGAGC  TTGGTATGAGGTATTGCTGG | 0.078 | 200 | SCC*mec* IVc | [15] |
| *mec* Type IVd-F  *mec* Type IVd-R | CTCAAAATACGGACCCCAATACA  TGCTCCAGTAATTGCTAAAG | 0.28 | 881 | SCC*mec* IVd | [15] |
| *mec* Type V-F  *mec* Type V-R | GAACATTGTTACTTAAATGAGCG  TGA AAGTTGTACCCTTGACACC | 0.06 | 325 | SCC*mec* V | [15] |
| [*arc*A_1](https://www.idtdna.com/orderstatus/SpecSheet.aspx?OrderNum=5150823&MfgID=82585206&MfgLocID=1&ProdID=1213&position=0)  [*arc*A_Chrom_2](https://www.idtdna.com/orderstatus/SpecSheet.aspx?OrderNum=5150823&MfgID=82584422&MfgLocID=1&ProdID=1213&position=1)  [*arc*A_ACME_3](https://www.idtdna.com/orderstatus/SpecSheet.aspx?OrderNum=5150823&MfgID=82584423&MfgLocID=1&ProdID=1213&position=2) | [CCWGGAAAAGAATTAGAAAAT](https://www.idtdna.com/orderstatus/SpecSheet.aspx?OrderNum=5150823&MfgID=82585206&MfgLocID=1&ProdID=1213&position=0)  TTGGATATCATCTATACC  [ACTTGTGTTTGCATCTTT](https://www.idtdna.com/orderstatus/SpecSheet.aspx?OrderNum=5150823&MfgID=82584423&MfgLocID=1&ProdID=1213&position=2) | 1.6  3.2  1.6 | 933  696 | Chrom-*arc*A  ACME-*arc*A | This study |
| Luk-PV-1  Luk-PV-2 | ATCATTAGGTAAAATGTCTGGACATGATCCA  GCATCAACTGTCTTGGATAGCAAAAGC | 0.4 | 433 | *pvl* | [16] |
| *cap*5-F  *cap*5-R | ATGACGATGAGGATAGCG  CTCGGATAACACCTGTTGC | 0.4 | 880 | *cap*5 | [17] |
